# Applied natural language processing in mental health big data

**DOI:** 10.1038/s41386-020-00842-1

**Published:** 2020-09-07

**Authors:** Robert Stewart, Sumithra Velupillai

**Affiliations:** 1 King’s College London, (Institute of Psychiatry, Psychology and Neuroscience), London, UK; 2 South London and Maudsley NHS Foundation Trust, London, UK

**Keywords:** Signs and symptoms, Medical research

‘Big data’ has transformative potential in mental health research, including the use of data from electronic health records and the ‘unlocking’ of text-field information contained here through natural language processing (NLP). Over the last 10 years, we have made substantial progress in applying NLP within the Clinical Record Interactive Search (CRIS) platform to enhance research at the South London and Maudsley Trust (SLaM): a large mental healthcare provider serving an urban catchment of around 1.3 million residents. CRIS provides a deidentified copy of SLaM’s electronic health record [1], accessed within a robust data security and governance framework, currently drawing data from over 500,000 patients and having supported over 200 published research papers. A number of other UK mental healthcare providers now have CRIS-like capability, extending the potential for multi-site projects.

‘First phase’ NLP on CRIS focused on capturing highest-priority constructs for research, hitherto ‘invisible’ within unstructured text. These included interventions received (e.g. medications, psychotherapies), indications for interventions (e.g. symptom profiles), and wider factors predicting intervention response and longer-term outcome (e.g. substance use, physical health comorbidity, educational achievement and occupation). Over 80 such ‘apps’ are detailed in a regularly updated online catalogue [2] and these have transformed the depth of data, and thus the range of investigations now possible without alterations required to clinician recording practice. This, for example, has enabled assessment of routine service outcomes against detailed text-derived symptomatic profiles hitherto unquantifiable at scale from a routine clinical record, such as negative syndrome in over 7500 patients with schizophrenia [3].

CRIS NLP development to date has largely involved the wide application of relatively straightforward techniques, principally clinical entity recognition, to address the main deficits in data extraction capability from the unmodified record. The next few years are likely to see more complex and technically ambitious innovations. Recent advances in NLP approaches, such as neural network models, allow the development of more robust extraction not only of additional clinical features, but also of more comprehensive entities from clinical text. Of particular interest are recent advances using so-called transformer models to generate contextual embeddings, which provide powerful language representations and require less annotation efforts for new clinical use-cases [4]. Other novel directions include moving beyond local clinical entities in documents to capture temporal information, for instance to identify the onset of psychotic symptoms [5] and thus capture ‘duration of untreated psychosis’ at scale, modelling complex entities from multiple keywords (such as experiences of violence or abuse), or applying NLP approaches that capture more context in the documents (such as the stereotyped paragraph sub-structure of clinical case summaries and the mental state examination). However, developing research environments where computational and clinical expertise is combined is crucial for these future innovations to have a real service impact. One interesting direction to reach a broader computational community is to use neural network-based NLP approaches inspired from machine translation methods to generate synthetic clinical text data, that can be accessed more widely for method development before deploying on real data [6]. Applied clinical NLP thus shows huge promise as a nascent specialty.

## Funding and declaration

RS and SV are part-funded by: (i) the National Institute for Health Research (NIHR) Biomedical Research Centre at the South London and Maudsley NHS Foundation Trust and King’s College London; (ii) a Medical Research Council (MRC) Mental Health Data Pathfinder Award to King’s College London. RS is additionally part-funded by (iii) an NIHR Senior Investigator Award; (iv) the National Institute for Health Research (NIHR) Applied Research Collaboration South London (NIHR ARC South London) at King’s College Hospital NHS Foundation Trust. The views expressed are those of the authors and not necessarily those of the NIHR or the Department of Health and Social Care. In the last 3 years, RS has received research support from Janssen, GSK and Roche. The authors have no conflicts of interest to declare in relation to the work described.
